# Prior caesarean section and likelihood of vaginal birth, 2012–2016, China

**DOI:** 10.2471/BLT.17.206433

**Published:** 2018-07-16

**Authors:** Yi Mu, Xiaohong Li, Jun Zhu, Zheng Liu, Mingrong Li, Kui Deng, Changfei Deng, Qi Li, Leni Kang, Yanping Wang, Juan Liang

**Affiliations:** a National Office for Maternal and Child Health Surveillance of China, Department of Obstetrics, West China Second University Hospital, Sichuan University, Chengdu, China.; b National Office for Maternal and Child Health Surveillance of China, Department of Paediatrics, West China Second University Hospital, Sichuan University, Chengdu, China.; c Key Laboratory of Birth Defects and Related Diseases of Women and Children (Sichuan University), Ministry of Education, Chengdu, China.

## Abstract

**Objective:**

To examine the trends and safety of vaginal birth after caesarean section around the period of the one-child policy relaxation in China.

**Methods:**

We used data from China's National Maternal Near Miss Surveillance System between 2012 and 2016. To examine trends in vaginal birth after caesarean section, we used Poisson regression with a robust variance estimator. We also assessed the association between vaginal birth after caesarean section and maternal and perinatal outcomes.

**Findings:**

We analysed 871 636 deliveries by women with a previous caesarean section. Both in 2012 and 2016, the rate of vaginal birth after caesarean section was 9.8%. After adjusting for institutional, sociodemographic and obstetric characteristics, the rate increased by 14% between 2012 and 2016 (adjusted relative risk, aRR: 1.14; 95% confidence interval, CI: 1.07–1.21). Compared to women with a repeat caesarean section, women with a vaginal birth after caesarean section experienced lower incidence of uterine rupture (aRR: 0.26, 95% CI: 0.16–0.42), blood transfusion (aRR: 0.68, 95% CI: 0.53–0.87) and admission to the intensive care unit (aRR: 0.36, 95% CI: 0.25–0.52), but higher incidence of intrapartum stillbirths, (aRR: 7.20, 95% CI: 6.09–8.51), newborns with a 5-minute Apgar score less than 7 (aRR: 1.75, 95% CI: 1.54–1.99) and neonatal death before discharge (aRR: 1.90, 95% CI: 1.61–2.24).

**Conclusion:**

Promotion of vaginal birth after caesarean section could increase the rate even further in China. To ensure the safety of mothers and their newborns, national policies and guidelines on vaginal birth after caesarean section are needed.

## Résumé

**Objectif:**

Examiner les tendances et la sûreté de l'accouchement vaginal après une césarienne autour de la période de l'assouplissement de la politique de l'enfant unique en Chine.

**Méthodes:**

Nous avons utilisé les données du Système de surveillance national des décès maternels évités de justesse de la Chine entre 2012 et 2016. Afin d'examiner les tendances relatives à l'accouchement vaginal après césarienne, nous avons utilisé une régression de Poisson et un estimateur de variance fiable. Nous avons également évalué le lien entre l'accouchement vaginal après césarienne et les résultats pour la santé maternelle et périnatale.

**Résultats:**

Nous avons analysé 871 636 accouchements de femmes ayant préalablement subi une césarienne. En 2012 et en 2016, le taux d'accouchements vaginaux après césarienne était de 9,8%. Après ajustement en fonction de caractéristiques institutionnelles, sociodémographiques et obstétriques, ce taux a augmenté de 14% entre 2012 et 2016 (risque relatif ajusté, RRa: 1,14; intervalle de confiance, IC, à 95%: 1,07-1,21). Comparées aux femmes ayant subi une césarienne itérative, les femmes accouchant par voie vaginale après une césarienne ont connu une plus faible incidence de rupture utérine (RRa: 0,26; IC à 95%: 0,16-0,42), de transfusion sanguine (RRa: 0,68; IC à 95%: 0,53-0,87) et d'admission dans une unité de soins intensifs (RRa: 0,36; IC à 95%: 0,25-0,52), mais une plus forte incidence de mortinaissances lors de l'accouchement (RRa: 7,20; IC à 95%: 6,09-8,51), de nouveau-nés avec un score d'Apgar à 5 minutes inférieur à 7 (RRa: 1,75; IC à 95%: 1,54-1,99) et de décès néonatals avant la sortie de l'hôpital (RRa: 1,90; IC à 95%: 1,61-2,24).

**Conclusion:**

La promotion de l'accouchement vaginal après césarienne pourrait faire progresser davantage encore le taux en Chine. Afin d'assurer la sécurité des mères et de leur nouveau-né, des politiques et des directives nationales sur l'accouchement vaginal après césarienne sont nécessaires.

## Resumen

**Objetivo:**

Examinar las tendencias y la seguridad del parto vaginal después de una cesárea durante el periodo de relajación de la política de un solo hijo en China.

**Métodos:**

Se usaron datos del Sistema Nacional de Vigilancia de Casi Accidentes Maternos de China (National Maternal Near Miss Surveillance System) de 2012 a 2016. Para examinar las tendencias del parto vaginal después de una cesárea, se utilizó la regresión de Poisson con un estimador de varianza robusto. También se evaluó la asociación entre el parto vaginal después de una cesárea y los resultados maternos y perinatales.

**Resultados:**

Se analizaron 871 636 partos de mujeres que ya habían pasado por una cesárea. Tanto en 2012 como en 2016, la tasa de partos vaginales después de una cesárea fue del 9,8%. Después de ajustar las características institucionales, sociodemográficas y obstétricas, la tasa aumentó un 14% entre 2012 y 2016 (coeficiente de riesgo ajustado, aRR: 1,14; intervalo de confianza, IC, del 95%: 1,07-1,21). En comparación con las mujeres que repitieron cesárea, las mujeres que tuvieron un parto vaginal después de una cesárea experimentaron una menor incidencia de rotura uterina (aRR: 0,26, IC del 95%: 0,16–0,42), transfusiones de sangre (aRR: 0,68, IC del 95%: 0,53–0,87) y hospitalización en la unidad de cuidados intensivos (aRR: 0,36, IC del 95%: 0,25–0,52), pero una mayor incidencia de mortinatos durante el parto, (aRR: 7,20, IC del 95%: 6,09–8,51), recién nacidos con una puntuación de Apgar de 5 minutos inferior a 7 (aRR: 1,75, IC del 95%: 1,54–1,99) y muerte neonatal antes del alta (aRR: 1,90, IC del 95%: 1,61-2,24).

**Conclusión:**

Promover el parto vaginal después de una cesárea podría aumentar aún más la tasa en China. Para garantizar la seguridad de las madres y los recién nacidos, son necesarias políticas y directrices nacionales sobre el parto vaginal después de una cesárea.

## ملخص

**الغرض:**

بحث اتجاهات وسلامة الولادة الطبيعية بعد ولادة قيصرية سابقة خلال فترة سياسة الطفل الواحد في الصين.

**الطريقة:**

لقد اعتمدنا على البيانات الواردة من النظام الوطني لمراقبة حالات نجاة الأمهات من الوفاة المؤكدة في الصين خلال الفترة من عام 2012 إلى عام 2016. ولبحث اتجاهات الولادة الطبيعية بعد الولادة القيصرية، فقد قمنا باستخدام تحليل التحوف لبواسون، مع مُقدّر قوي للتباين. كما قمنا أيضا بتقييم الارتباط بين الولادة الطبيعية بعد الولادة القيصرية، والنتائج المترتبة على فترة الأمومة والولادة.

**النتائج:**

قمنا بتحليل 871636 حالة ولادة لسيدات كان لهم تجربة سابقة مع الولادة القيصرية. خلال عامي 2012 و2016، كان معدل الولادة الطبيعية بعد الولادة القيصرية هو 6.8%. وبعد تعديل الخصائص المؤسسية والاجتماعية والديموغرافية والمتعلقة بالولادة، زاد المعدل بنسبة 14% بين عامي 2012 و2016 (نسبة المخاطر المعدلة، aRR: 1.14؛ فاصل الثقة 95%: 1.07–1.21). ومقارنة بالنساء اللاتي خضعن لعملية الولادة القيصرية عدة مرات، فإن النساء اللاتي خضعن للولادة الطبيعية بعد ولادة قيصرية سابقة، شهدن حالات أقل لتمزق الرحم (نسبة المخاطر المعدلة: 0.26، فاصل الثقة 95%: 0.16 إلى 0.42)، ونقل الدم (نسبة المخاطر المعدلة: 0.68، فاصل الثقة 95%: 0.53 إلى 0.87) ودخول وحدة العناية المركزة (نسبة المخاطر المعدلة: 0.36، فاصل الثقة 95%: 0.25 إلى 0.52)، ولكن كان هناك ارتفاع في حالات وفاة الأطفال أثناء الولادة، (نسبة المخاطر المعدلة: 7.20، فاصل الثقة 95%: 6.09 إلى 8.51)، وسجل المواليد الجدد درجة أقل من 7 في فحص Apgar بعد مرور 5 دقائق من الولادة (نسبة المخاطر المعدلة: 1.75، فاصل الثقة 95%: 1.54 إلى 1.99) ووفاة حديثي الولادة قبل الخروج من المستشفى (نسبة المخاطر المعدلة: 1.90، فاصل الثقة 95%: 1.61 إلى 2.24).

**الاستنتاج:**

يمكن أن يؤدي الترويج للولادة الطبيعية بعد الولادة القيصرية إلى زيادة المعدل أكثر من ذلك في الصين. لضمان سلامة الأمهات ومواليدهم، فإن هناك حاجة إلى سياسات وتوجيهات وطنية تنظم الولادة الطبيعية بعد الولادة القيصرية. ****

## 摘要

**目的:**

旨在探讨中国独生子女政策放宽期间，剖腹产术后阴道分娩的趋势和安全性。

**方法:**

我们使用从中国危重孕产妇监测系统中获取的 2012 至 2016 年间的数据。为了检查剖腹产术后阴道分娩的趋势，使用带有稳定方差估计值的 Poisson 回归系统。我们还评估了剖腹产术后阴道分娩与产妇妊娠结局和围产儿结局之间的关系。

**结果:**

我们分析了有剖腹产史的妇女的 871636 次分娩情况。在 2012 和 2016 年，剖腹产术后阴道分娩率均为 9.8％。在调整了机构、社会人口和产科特征后，2012 年至 2016 年期间，剖腹产术后阴道分娩率上升了 14％（调整后的相对风险，aRR：1.14；95% 置信区间，CI：1.07-1.21）。与重复剖腹产妇女相比，剖腹产术后阴道分娩的妇女，子宫破裂（aRR：0.26; 95% CI：0.16-0.42）、输血（aRR：0.68; 95% CI：0.53-0.87）和入住重症监护室（aRR：0.36; 95% CI：0.25-0.52）的发生率较低，但分娩时死胎（aRR：7.20; 95% CI：6.09-8.51）、新生儿 5 分钟 Apgar 评分低于 7 分（aRR：1.75; 95% CI：1.54-1.99）和出院前新生儿死亡（aRR：1.90; 95% CI：1.61-2.24）的发生率较高。

**结论:**

促进剖腹产术后阴道分娩，可以进一步提高中国境内的阴道分娩率。为确保母亲及其新生儿的安全，需要有关剖腹产术后阴道分娩的国家政策和指导方针。****

## Резюме

**Цель:**

Изучить тенденции и оценить степень безопасности естественных родов после кесарева сечения в период смягчения политики Китая «Одна семья — один ребенок».

**Методы:**

В период с 2012 по 2016 годы авторы использовали данные Национальной системы надзора за беременными женщинами в Китае. При исследовании тенденций естественных родов после кесарева сечения использовалась регрессия Пуассона с устойчивой оценкой дисперсии. Авторы также оценили связь между естественными родами после кесарева сечения и исходами беременности и родов.

**Результаты:**

Был проанализирован исход 871 636 родов у женщин с предшествующим кесаревым сечением. Как в 2012 году, так и в 2016 году частота естественных родов после кесарева сечения составила 9,8%. После учета поправок на институциональные, социально-демографические и акушерские характеристики показатель увеличился на 14% в период между 2012 и 2016 годами (скорректированный относительный риск, сОР: 1,14; 95%-й доверительный интервал, ДИ: 1,07–1,21). По сравнению с женщинами с повторным кесаревым сечением у женщин с естественными родами после кесарева сечения была более низкая частота возникновения разрыва матки (сОР: 0,26; 95%-й ДИ: 0,16–0,42), необходимости переливания крови (сОР: 0,68; 95%-й ДИ: 0,53–0,87) и перевода в палату реанимации и интенсивной терапии (сОР: 0,36; 95%-й ДИ: 0,25–0,52), но более высокая частота интранатальной смерти плода (сОР: 7,20; 95%-й ДИ: 6,09–8,51), рождения младенцев с оценкой по шкале Апгар менее семи баллов через пять минут после рождения (сОР: 1,75; 95%-й ДИ: 1,54–1,99) и смерти новорожденных до выписки (сОР: 1,90; 95%-й ДИ: 1,61–2,24).

**Вывод:**

Популяризация естественных родов после кесарева сечения может еще больше увеличить эти показатели в Китае. Для обеспечения безопасности матерей и новорожденных необходима национальная политика и рекомендации для практики естественных родов после кесарева сечения.****

## Introduction

Caesarean sections can save maternal, fetal and neonatal lives, but overuse of caesarean sections is a threat to short and long-term maternal and child health.^1^^–^^3^ The recent increase in caesarean section rate worldwide has led to global concerns^4^^–^^6^ and experts have suggested vaginal birth after caesarean section as an effective way to reduce caesarean section rate.^7^^,^^8^ Since both elective repeat caesarean section and vaginal birth after caesarean section is associated with both benefits and risks for pregnant women with a previous caesarean section,^9^^–^^11^ academic organizations of obstetrics and gynaecology have reached a consensus on when and how to offer vaginal birth after caesarean section.^12^^,^^13^

In China, the caesarean section rate increased from 29% in 2008 to 35% in 2014.^14^ A survey of nine Asian countries in 2010 showed that China had the highest caesarean section rate,^15^ which prompted the Chinese government to establish several policies to reduce the rate.^14^^,^^16^^,^^17^ These policies mostly targeted women without a previous caesarean section. A recent Chinese study reported that the caesarean section rate decreased from 45.3% in 2012 to 41.1% in 2016, mainly due to reduction in women targeted by the policies.^18^ During the one-child policy in China, hospitals were generally reluctant to try vaginal birth in women with a previous caesarean section. Pregnant women and their families also preferred to have a repeat caesarean section. However, the proportion of pregnant women with a previous caesarean section increased rapidly after the relaxation of the one-child policy in November 2013.^18^^,^^19^ Hence vaginal birth after caesarean section seemed as an important option to reduce caesarean section rate.

A few studies in China have examined vaginal birth after caesarean section.^20^^–^^22^ However, they were conducted in not more than five hospitals and no national representative study has been published. Reports on policy needs and maternal and perinatal outcomes for such delivery are also lacking.

Here, we used data from 438 health facilities across China to assess the current situation of vaginal birth after caesarean section. We compare the maternal and perinatal outcomes between vaginal birth after caesarean section and repeat caesarean section to identify whether vaginal birth after caesarean section can be a safe delivery alternative. We also provide several suggestions for further promotion of vaginal birth after caesarean section in China.

## Methods

### Data

We obtained individual level data from 1 January 2012 and 31 December 2016 and institutional data for the year 2015 from China's National Maternal Near Miss Surveillance System.

The surveillance system was established in October 2010 and covers 441 health facilities at county level or above from 326 urban districts or rural counties in 30 provinces in China. The system enumerates all maternal deaths and near misses, that is, women who nearly died from a severe complication of pregnancy or delivery, using the same approach suggested in the World Health Organization’s (WHO) global survey on maternal and perinatal health.^15^ The system oversample large hospitals in urban districts, because some urban districts and rural counties do not have hospitals with the required number of births, that is, more than 1000 deliveries per year. Sampling strategy, data collection and reporting processes and quality control method of surveillance system have been detailed elsewhere.^18^^,^^23^ We obtained de-identified data.

The system collected data on each health facility in 2015, including the location of the facility (east, central or west China and urban or rural location), the health facility level (levels 1 to 3, based on the number of beds, categories of clinical departments, numbers of medical personnel, type and quantity of equipment and hospital funding, where level-1 consist of the smallest hospitals and level-3 the largest)^24^ and the number of obstetricians.

We excluded three health facilities that did not report any data after 2012. We included only singleton pregnant women with at least one previous caesarean section and who delivered at or after 28 completed weeks of gestation or with a newborn’s birth weight of at least 1000 g.

We classified maternal age and the number of antenatal care visits into five groups and used common definitions for marital status and education as detailed elsewhere.^18^^,^^23^ For the institutional data, we calculated the number of obstetricians per 1000 births using the number of births reported in the surveillance system in 2015. We extracted the day of the week from the date of delivery.

### Statistical analysis

As a result of the oversampling of large urban hospitals, we were unable to access data on the distribution of deliveries per hospital in each region and urban or rural area. Therefore, we report the rates of vaginal birth after caesarean section by weighting for the sampling distribution of the population according to the 2010 census of China. The method is detailed elsewhere.^18^^,^^23^

We used *χ^2^* test to assess the trends in vaginal birth after caesarean section by maternal age, number of previous caesarean sections and hospital level. We also calculated the trends in the weighted rates of vaginal birth after caesarean section in each subgroup.

To examine the association between year and the rate of vaginal birth after caesarean section, we used Poisson regression with a robust variance estimator, since we were analysing rare events and the we had binomial data on individuals.^25^ We used STATA version 15.0 (StataCorp. Lp, College Station, United States of America) for our analyses.^26^ For assessing trends for all births and for the groups: maternal age, number of previous caesarean sections and hospital level, we calculated the crude relative risk (cRR) and 95% confidence intervals (CI) by weighting the sampling distribution of the population and clustering of births within hospitals. We also calculated the adjusted RR (aRR) and 95% CI by further adjusting for institutional factors (region, hospital level, number of obstetricians per 1000 births, day of the week), individual sociodemographic characteristics (number of antenatal care visits, education, marital status, maternal age) and clinical factors that may be associated with vaginal birth after caesarean section (number of previous caesarean sections, gestational age and newborn’s birth weight). We investigated both multicollinearity and model goodness-of-fit to identify the most robust and stable model. We used a likelihood ratio test to obtain the *P*-value for the interaction among the variables by comparing models with and without interaction variables.

Lastly, we compared the proportions of maternal and neonatal outcomes between vaginal birth after caesarean section and repeat caesarean section. We calculated the cRR and aRR for the following maternal outcomes: uterine rupture, blood transfusion, hysterectomy, intensive care unit admission and mortality before discharge; and perinatal outcomes: intrapartum stillbirths, 5-minute Apgar score lower than 7 and neonatal deaths before discharge. We restricted the sample to births without antepartum stillbirths for intrapartum stillbirths; to live births for 5-minute Apgar score lower than 7; and neonatal deaths before discharge. We defined uterine rupture as uterine or lower uterine dehiscence in late pregnancy or during childbirth, including complete and incomplete rupture.^27^

## Results

Between 2012 and 2016, 871 636 singleton births from 438 hospitals met the inclusion criteria. Of these, 82 778 women had a vaginal birth, giving a weighted rate for vaginal birth after caesarean section of 9.6%. The weighted rates of vaginal birth were stable over the study period; the rates were 9.8% in both 2012 and in 2016 (Table 1). The proportion of women older than 29 years giving birth increased from 49.9% (58 066/116 471) in 2012 to 58.0% (142 798/246 161) in 2016 (Table 2), while the weighted rates of vaginal birth after caesarean section only increased for women younger than 35 years (Table 1). The proportion of women with two previous caesarean sections increased from 4.3% (5263/121 692) to 5.2% (13 111/251 069; Table 2). However, the weighted rate of vaginal birth after caesarean section only increased for women with one previous caesarean section, from 9.7% in 2012 to 9.9% in 2016. In 2012 and 2013, the weighted rates of vaginal birth were higher in women with at least two previous caesarean sections than women with one previous section (Table 1). The proportion of women who delivered in level-3 hospitals increased from 34.9% (42 465/121 692) to 42.3% (106 313/251 069; Table 2) and was consistent with an increase in the weighted vaginal birth rate, from 7.9% in 2012 to 9.1% in 2016.

**Table 1:** Weighted rates of vaginal birth after caesarean section, by maternal age, number of previous caesarean sections and hospital level, China, 2012–2016

Characteristic	Weighted % of vaginal birth after caesarean section^a^				
	2012	2013	2014	2015	2016
**Maternal age, years**					
< 20	17.4	16.8	15.0	15.8	20.0
20–24	11.6	11.2	11.5	12.9	13.6
25–29	10.1	9.7	9.4	10.2	10.5
30–34	8.4	8.2	8.0	8.7	8.9
≥ 35	8.6	7.7	7.5	8.0	8.0
**No. of previous caesarean sections**					
1	9.7	9.3	9.2	9.8	9.9
2	12.7	10.6	8.9	8.8	8.2
≥ 3	14.3	11.2	11.8	10.7	5.8
**Hospital level**					
Level 1	9.1	7.6	7.3	7.7	8.2
Level 2	10.6	10.2	9.8	10.3	10.0
Level 3	7.9	7.5	7.8	8.6	9.1
Unknown	12.5	12.2	13.2	13.3	16.0
**All births**	9.8	9.3	9.1	9.7	9.8

^a^ Weighted for sampling distribution of the population.

Notes: We obtained data from 438 hospitals. Level-1 hospitals are the smallest hospitals, while level-3 hospitals are the largest.

**Table 2:** Trends in the distribution of births by women with a previous caesarean section, China, 2012–2016

Characteristic	No. of births (%)					*P*^a^
	2012	2013	2014	2015	2016	
**Maternal age, years^b^**						
< 20	480 (0.4)	599 (0.5)	759 (0.4)	877 (0.5)	890 (0.4)	0.00
20–24	14 654 (12.6)	15 638 (12.2)	18 035 (10.5)	16 850 (9.2)	17 402 (7.1)	
25–29	43 271 (37.2)	46 227 (36.1)	63 751 (37.3)	65 773 (35.9)	85 071 (34.6)	
30–34	39 488 (33.9)	43 703 (34.1)	59 761 (34.9)	64 258 (35.1)	91 888 (37.3)	
≥ 35	18 578 (16.0)	21 999 (17.2)	28 795 (16.8)	35 318 (19.3)	50 910 (20.7)	
**No. of previous caesarean sections**						
1	116 352 (95.6)	125 789 (95.1)	167 920 (94.7)	178 118 (94.1)	237 616 (94.6)	0.00
2	5 263 (4.3)	6 316 (4.8)	9 256 (5.2)	10 851 (5.7)	13 111 (5.2)	
≥ 3	77 (0.1)	130 (0.1)	207 (0.1)	288 (0.2)	342 (0.1)	
**Hospital level**						
Level 1	10 546 (8.7)	10 937 (8.3)	12 669 (7.1)	12 907 (6.8)	15 716 (6.3)	0.00
Level 2	63 376 (52.1)	67 971 (51.4)	87 968 (49.6)	90 080 (47.6)	116 842 (46.5)	
Level 3	42 465 (34.9)	47 439 (35.9)	68 571 (38.7)	78 294 (41.4)	106 313 (42.3)	
Unknown	5 305 (4.4)	5 888 (4.5)	8 175 (4.6)	7 976 (4.2)	12 198 (4.9)	
**All births**	121 692 (100.0)	132 235 (100.0)	177 383 (100.0)	189 257 (100.0)	251 069 (100.0)	NA

NA: not applicable.

^a^ We used *X^2^*-test to examine the distribution differences.

^b^ Information about maternal age were missing from 5221 birth records in 2012, 4069 records in 2013, 6282 records in 2014, 6181 records in 2015 and 4908 records in 2016. In total 26661 (3.1%) records were missing information about maternal age.

Notes: We obtained data from 438 hospitals. We included only singleton pregnant women with at least one previous caesarean section and delivered at or after 28 completed weeks of gestation or with a newborn’s birth weight of at least 1000 g. Level-1 hospitals are the smallest hospitals, while level-3 hospitals are the largest. Inconsistencies arise in some values due to rounding.

There was no change in the crude rates of vaginal birth after caesarean section between 2012 and 2016 (cRR: 1.00; 95% CI: 0.93–1.07). However, after adjusting for confounding factors, the vaginal birth rate increased by 14% (aRR: 1.14; 95% CI: 1.07–1.21; Table 3). We found increasing trends for women older than 20 years, for women with one previous caesarean section and in level-3 hospitals. The rate decreased in women with two or more previous caesarean sections.

**Table 3:** Trends in vaginal birth after caesarean section by maternal age, number of previous caesarean sections and hospital level, China, 2012–2016

Characteristic	Vaginal birth after caesarean section											
	cRR (95% CI)					*P*^a^	aRR (95% CI)					*P*^a^
	2012	2013	2014	2015	2016		2012	2013	2014	2015	2016	
**Maternal age, years^b^**												
< 20	1.00	0.97 (0.73–1.27)	0.87 (0.66–1.13)	0.91 (0.71–1.17)	1.15 (0.90–1.47)	0.0000	1.00	1.05 (0.82–1.36)	0.91 (0.71–1.16)	0.95 (0.75–1.21)	1.17 (0.94–1.47)	0.0046
20–24	1.00	0.97 (0.89–1.05)	0.99 (0.92–1.07)	1.11 (1.02–1.22)	1.18 (1.07–1.29)		1.00	0.95 (0.88–1.03)	0.99 (0.92–1.07)	1.10 (1.01–1.19)	1.15 (1.05–1.26)	
25–29	1.00	0.96 (0.91–1.01)	0.93 (0.87–1.00)	1.00 (0.92–1.09)	1.04 (0.95–1.13)		1.00	0.95 (0.90–1.00)	0.96 (0.90–1.02)	1.04 (0.96–1.13)	1.12 (1.03–1.21)	
30–34	1.00	0.97 (0.91–1.03)	0.95 (0.88–1.02)	1.04 (0.95–1.13)	1.06 (0.97–1.16)		1.00	0.98 (0.92–1.03)	1.01 (0.95–1.08)	1.13 (1.04–1.22)	1.20 (1.11–1.29)	
≥ 35	1.00	0.90 (0.83–0.97)	0.88 (0.81–0.95)	0.94 (0.85–1.03)	0.93 (0.85–1.02)		1.00	0.92 (0.85–0.98)	0.96 (0.89–1.04)	1.07 (0.98–1.16)	1.11 (1.02–1.21)	
**No. of previous caesarean sections^c^**												
1	1.00	0.95 (0.92–0.99)	0.94 (0.89–0.99)	1.00 (0.94–1.07)	1.02 (0.95–1.10)	0.0000	1.00	0.96 (0.92–1.00)	1.00 (0.95–1.05)	1.09 (1.02–1.16)	1.17 (1.09–1.25)	0.0000
2	1.00	0.84 (0.71–1.00)	0.70 (0.58–0.85)	0.69 (0.58–0.83)	0.65 (0.55–0.77)		1.00	0.87 (0.75–1.02)	0.78 (0.66–0.93)	0.79 (0.66–0.93)	0.74 (0.63–0.87)	
≥ 3	1.00	0.78 (0.36–1.70)	0.83 (0.38–1.79)	0.75 (0.34–1.64)	0.41 (0.21–0.81)		1.00	0.68 (0.32–1.44)	0.76 (0.36–1.59)	0.76 (0.35–1.62)	0.47 (0.25–0.88)	
**Hospital level^d^**												
Level 1	1.00	0.83 (0.74–0.93)	0.79 (0.68–0.93)	0.84 (0.70–1.02)	0.90 (0.71–1.14)	0.0000	1.00	0.81 (0.72–0.92)	0.79 (0.68–0.91)	0.84 (0.70–1.02)	0.91 (0.73–1.13)	0.0000
Level 2	1.00	0.96 (0.91–1.01)	0.92 (0.86–0.99)	0.97 (0.90–1.05)	0.94 (0.86–1.02)		1.00	0.98 (0.93–1.03)	0.98 (0.92–1.04)	1.05 (0.98–1.14)	1.08 (1.00–1.17)	
Level 3	1.00	0.95 (0.90–1.01)	0.99 (0.89–1.09)	1.09 (0.96–1.25)	1.15 (1.03–1.30)		1.00	0.93 (0.88–0.99)	1.02 (0.94–1.11)	1.16 (1.04–1.29)	1.28 (1.16–1.41)	
Unknown	1.00	0.98 (0.87–1.10)	1.06 (0.92–1.22)	1.06 (0.89–1.27)	1.28 (0.89–1.84)		1.00	0.92 (0.84–1.01)	1.01 (0.92–1.10)	0.97 (0.85–1.12)	1.26 (0.93–1.71)	
**Total**	1.00	0.95 (0.91–0.98)	0.93 (0.88–0.98)	0.98 (0.92–1.05)	1.00 (0.93–1.07)	NA	1.00	0.95 (0.92–0.99)	0.98 (0.93–1.03)	1.07 (1.00–1.14)	1.14 (1.07–1.21)	NA

aRR: adjusted relative risk; CI: confidence interval; cRR: crude relative risk; NA: not applicable.

^a^ We calculated *P*-value for interactions between year and maternal age; year and number of previous caesarean sections; and year and hospital level.

^b^ We did not adjust for maternal age in the age groups.

^c^ We did not adjust for number of caesarean sections in previous pregnancy when calculating aRR for number of previous caesarean sections.

^d^ We did not adjust for hospital level in the hospital-level group.

After adjusting for confounding factors, we found that women with a vaginal birth after caesarean section had lower incidence of uterine rupture (aRR: 0.26; 95% CI: 0.16–0.42), blood transfusion (aRR: 0.68; 95% CI: 0.53–0.87) and fewer admissions to the intensive care units (aRR: 0.36; 95% CI: 0.25–0.52) than women with repeated caesarean sections. We observed no differences in the incidences of hysterectomy and maternal mortality before discharge between the two groups. Women with vaginal birth after caesarean section had an approximately 19-fold higher incidence in intrapartum stillbirths, and neonates had a threefold higher incidence of 5-minute Apgar score less than 7 and a fourfold higher incidence of neonatal death before discharge than the repeat caesarean section group. These differences persisted even after adjustment for confounding factors (intrapartum stillbirths aRR: 7.20, 95% CI: 6.09–8.51; Apgar score aRR: 1.75, 95% CI: 1.54–1.99; of neonatal deaths aRR: 1.90, 95% CI: 1.61–2.24; Table 4).

**Table 4:** Comparison of maternal and perinatal outcome between vaginal birth after caesarean section and repeat caesarean section, China, 2012–2016

Outcome	Weighted rate (no. of births)^a^		cRR (95% CI)^d^	aRR (95% CI)^e^
	Vaginal birth after caesarean section^b^	Repeat caesarean section^c^		
**Maternal**				
No. of uterine ruptures per 1 000 births	2.1 (195)	6.5 (6 415)	0.32 (0.21–0.50)	0.26 (0.16–0.42)
No. of blood transfusions per 1 000 births	10.0 (814)	14.4 (10 780)	0.69 (0.59–0.82)	0.68 (0.53–0.87)
No. of hysterectomies per 1 000 births	0.5 (39)	0.4 (288)	1.28 (0.82–2.02)	0.78 (0.45–1.32)
No. of ICU admissions per 1 000 births	1.2 (134)	3.1 (3 595)	0.39 (0.24–0.63)	0.36 (0.25–0.52)
Maternal mortality before discharge per 100 000 births	7.6 (9)	3.5 (33)	2.15 (1.08–4.27)	1.26 (0.55–2.90)
**Perinatal**				
No. of intrapartum stillbirths per 1 000 births^e^	8.8 (801)	0.5 (401)	19.18 (15.5–23.73)	7.20 (6.09–8.51)
No. of 5-minute Apgar scores < 7 per 1 000 live births^f^	8.4 (706)	2.8 (2 230)	3.00 (2.43–3.70)	1.75 (1.54–1.99)
No. of neonatal deaths before discharge per 1 000 live births^g^	3.4 (297)	0.8 (649)	4.31 (3.54–5.26)	1.90 (1.61–2.24)

aRR: adjusted relative ratio; CI: confidence interval; cRR: crude relative ratio; ICU: intensive care unit.

^a^ No. of births represents all births if not indicated live births.

^b^ The sample for vaginal birth after caesarean section were 82 778 births and 77 826 live births.

^c^ The sample for repeat caesarean sections were 788 858 births and 787 371 live births.

^d^ We weighted the rate for sampling distribution of population and adjustment for clustering of births within hospitals.

^e^ We adjusted the cRR for year, region, hospital level, number of obstetricians, day of week, number of antenatal care visits, education and marital status, maternal age, number of caesarean section in previous pregnancy, gestational age and birth weight.

^f^ Information about perinatal outcome was missing from 62 (0.01%) births. We excluded 5175 (0.59%) antepartum stillbirths from the total number of births.

^g^ We restricted the analysis to live births.

## Discussion

Our study shows that the adjusted rate of vaginal birth after caesarean section rate increased during the relaxation of the one-child policy. We found increasing trends for women older than 20 years, for women with one previous caesarean section and in level-3 hospitals. Compared to the group with a repeat caesarean section, the group with a vaginal birth after caesarean section had lower incidence of maternal adverse outcome, but higher incidence of perinatal adverse outcome.

A study reported that between 2012 and 2016, the proportion of pregnant women with a previous caesarean section in China increased from 9.8% (128 107/1 309 713) to 17.7% (265 770/1 503 630).^18^ This could be explained by the large number of women with a previous caesarean section and the increase in number of women wanting to have a second child. The National Bureau of Statistics of China reported that in 2017, 51.2% (8 830 000/17 230 000) of births were a birth of a second child.^28^ This data suggests that the repeat caesarean section rate will increase in China, if no measures are taken. Studies from several countries have shown that vaginal birth after caesarean section is an effective measure for reducing repeat caesarean section rates.^7^^,^^13^^,^^29^^–^^33^ Despite the debate in China whether to support vaginal birth after caesarean section as a measure to reduce the caesarean section rate,^34^^,^^35^ hospitals at all levels have encouraged trial of labour after caesarean section since 2010. This promotion has been done to achieve reduction in the caesarean section rate and to meet the demand from pregnant women with a prior caesarean section wanting a vaginal birth. In recent years, hospitals with advanced medical equipment and obstetric skills have increased the number of vaginal births after caesarean section [Liang J, et al., National Office for Maternal and Child Health Surveillance of China, unpublished data, 25 June 2018], which is consistent with our findings of an increased rate in level-3 hospitals.

The results from our large nationally representative study make it possible to compare the Chinese trends with other countries. Compared with Canada and the United States of America, which have had rates of 33.4% and 28.3%, respectively,^30^^,^^31^ the rate in China seems to have a potential to further increase. However, women in China with a previous caesarean section still have limited choices for a preferred method of delivery. For example, the cost of caesarean section is twice as high as the cost of vaginal deliveries,^18^ regardless of the previous delivery mode. Furthermore, obstetricians both have to bear the risk of being sued as the result of an adverse outcome and bear the loss of income. Hence, obstetricians may be reluctant to implement vaginal birth after caesarean section in their wards.

Although vaginal birth after caesarean section contributed to reducing repeat caesarean section rate in many countries, several problems have been reported with such delivery mode. For example, increasing risk of uterine rupture, operative injury, fetal or neonatal death and providers’ fear of liability.^36^^–^^38^ Subsequently, the vaginal birth after caesarean section rate has decreased in many countries.^32^^,^^33^^,^^39^ In our study, we found a higher incidence of perinatal adverse outcomes in the group of women with vaginal birth after caesarean section than for the group with repeat caesarean section, which is in contrast to other studies.^8^^,^^40^^,^^41^ This could be explained by the lack of guidelines in China before 2016, which may have led to inadequate screening of suitable women and their fetuses for vaginal birth after caesarean section in some hospitals. This lack of guidance might also explain why the proportion of women with two or more previous caesarean sections undergoing vaginal birth was nearly 5%. Furthermore, up to the end of 2015, the proportion for this group was higher than for women with one previous caesarean section. In August 2016, the Chinese Society of Obstetrics and Gynaecology issued an expert consensus on indications and contraindications of trial of labour after caesarean section, prenatal health education on pregnancy after caesarean section, and clinical guidance on implementing vaginal birth after caesarean section.^42^

The lower rate of vaginal birth after caesarean section in China, compared with other countries and higher incidence of perinatal adverse outcome, suggest that the current Chinese health system may not be equipped to provide delivery options for pregnant women with a previous caesarean section. However, vaginal birth after caesarean section is beneficial for the rational use of constrained medical resources and for the reduction of secondary trauma for women with a previous caesarean section. Therefore, promoting vaginal birth after caesarean section in China and ensuring the safety of mothers and newborns is needed. We have the following suggestions for promoting this delivery mode.

First, policies need to be established and implemented at the national level. The health administration department could recommend: (i) hospitals that carry out vaginal birth after caesarean section, establish a dedicated delivery room for trial of labour after caesarean section, where emergency caesarean section is possible; (ii) that a curriculum for vaginal birth after caesarean section is added to the midwifery training; (iii) hospitals to revise the fee for vaginal birth after caesarean section and increase the income of obstetricians who implement this form of delivery; and (iv) introduction of public health education on vaginal birth after caesarean section.

Second, the clinical guidelines for vaginal birth after caesarean section could be improved. The expert consensus issued in 2016^42^ was written only by obstetrics experts and lacked the involvement of perinatal experts, especially neonatal experts. Therefore, the consensus has less guidance on how to deal with perinatal adverse outcomes from vaginal birth after caesarean section. Furthermore, an article recently pointed out that the evidence used by the expert consensus was insufficient and the quality of the consensus was doubtful, due to lack of a formal approach.^43^ Therefore, the expert consensus needs to be revised and the obstetrics and neonatal academic associations should jointly issue formal clinical guidelines. We suggest that the revised guidelines should include: (i) labour standard for stage of labour specifically for trial of labour after caesarean section, since the new definition of stage of labour introduced in China are not suitable for trial of labour after caesarean section;^44^ and (ii) involve neonatologists in the process of trial of labour after caesarean section, to ensure the timely treatment for perinatal adverse outcome.

Our study has several limitations. First, as the surveillance system oversampled large hospitals located in urban areas and we only weighted the data for the population distribution in each region according to the 2010 census. Whether this kind of weighting method could solve the oversampling issue is unknown. Second, we could not obtain the number of women undergoing trial of labour after caesarean section or elective repeat caesarean section. Therefore, there might be a bias in the comparison of outcomes between vaginal birth after caesarean sections and repeat caesarean sections. For example, a woman having a uterine rupture during trial of labour after caesarean section will receive an urgent caesarean section and the delivery will be classified as a repeat caesarean section. Two Chinese studies reported that the success rate of trial of labour after caesarean section was about 70% and that nearly 10% of the failure was due to uterine rupture during the trial of labour.^22^^,^^45^ Thus, we hypothesize that the incidence of uterine rupture might be higher in trial of labour after caesarean section than vaginal birth after caesarean section, and the incidence of uterine rupture might be lower for women with an elective repeat caesarean section than women with a repeat caesarean section. Inconsistencies exist in the reported association between maternal outcome and mode of delivery when comparing trial of labour or vaginal birth after caesarean section to (elective) repeat caesarean section.^8^^,^^11^^,^^46^^–^^48^ Furthermore, systematic reviews have not identified any published randomized controlled trials,^9^^,^^49^ indicating a lack of robust evidence to confirm the benefits and harms of both repeat caesarean sections and trial of labour or vaginal birth after caesarean section.

In conclusion, the change in fertility policy offers an opportunity to promote vaginal birth after caesarean section in China. The increase of vaginal birth after caesarean section could contribute to the continued decline of the caesarean section rate in the country. Therefore, after the implementation of the two-child policy in October 2016, new policies and clinical supports are needed to provide a functioning environment for implementing vaginal birth after caesarean section. Policies for this form of delivery should not completely copy the past policies for reducing the caesarean section rate in China, such as setting a target rate, because of the risks associated with vaginal birth after caesarean section. Furthermore, good communication between the health-care provider and the woman is important when choosing the mode of delivery and that they take into consideration both the maternal and fetal conditions to ensure the mother’s and the fetus’ safety. These suggested actions will change the current choice of the mode of delivery for women with more than one pregnancy in China.
